# Health Literacy and Chronic Disease Self-Efficacy Among Older Adults with Multimorbidity: Evidence from Low-Resource Context

**DOI:** 10.3390/healthcare14152294

**Published:** 2026-07-29

**Authors:** Velide Pulomemoj, Anisa Subashi, Rinalda Proko, Lorena Serjanaj, Fatjona Kamberi

**Affiliations:** 1 Department of Biomedicine and Prevention, University of Rome Tor Vergata, 00133 Rome, Italy; 2 Faculty of Social Sciences, University of Tirana, 1010 Tirana, Albania; anisasubashi@gmail.com; 3 Independent Researcher, Newport News, VA 23601, USA; rinaldaproko@gmail.com; 4 Department of Health and Social Care, CECOS College, London E1 4NQ, UK; lorenaalikaj@yahoo.com; 5 Scientific Research Center for Public Health, Faculty of Health, University of Vlore “Ismail Qemali”, 9400 Vlora, Albania; fatjona.kamberi@univlora.edu.al

**Keywords:** health literacy, self-efficacy, older adults, multimorbidity, chronic diseases, low-resource settings, primary healthcare

## Abstract

**Highlights:**

**What are the main findings?**

- Older adults with multimorbidity in primary healthcare settings in Albania showed a high prevalence of limited health literacy, which remained an independent predictor of chronic disease self-efficacy after adjusting for demographic and clinical characteristics.
- Higher health literacy, education, and self-rated health were associated with greater chronic disease self-efficacy, while both the HLS-EU-Q16 and CDSES-33 demonstrated excellent internal consistency.

**What are the implications of the main findings?**

- The findings highlight the importance of integrating health literacy into chronic disease management for older adults with multimorbidity in primary healthcare.
- Health-literacy-responsive interventions may strengthen chronic disease self-efficacy and support more effective self-management among older adults, particularly in low-resource settings.

**Abstract:**

**Background/Objectives:** Multimorbidity among older adults is increasing globally and places significant pressure on primary healthcare systems, particularly in low-resource settings. Health literacy is recognized as an important determinant of chronic disease management; however, evidence regarding its association with chronic disease self-efficacy remains limited. This study examined the association between health literacy and chronic disease self-efficacy among older adults with multimorbidity receiving primary healthcare services in Albania. **Methods:** A cross-sectional study was conducted among 464 adults aged ≥65 years with at least two chronic conditions attending primary healthcare centres in Vlora Municipality. Stratified random sampling was used. Data were collected using a sociodemographic questionnaire, the European Health Literacy Questionnaire (HLS-EU-Q16), and the Stanford Chronic Disease Self-Efficacy Scale (CDSES-33). Health literacy was classified into three categories: inadequate, problematic, or adequate. Pearson’s correlation and hierarchical multiple linear regression analyses were performed after adjustment for relevant sociodemographic and clinical characteristics. **Results:** Participants were predominantly women (54.3%), with a mean age of 69.9 ± 4.7 years. Hypertension (72.6%) and diabetes mellitus (70.3%) were the most prevalent chronic conditions. Health literacy demonstrated a moderate positive correlation with chronic disease self-efficacy (*r* = 0.407, *p* < 0.001). In the fully adjusted regression model, health literacy remained an independent predictor of chronic disease self-efficacy. Higher educational attainment and better self-rated health were also significantly associated with greater chronic disease self-efficacy. **Conclusions:** Older adults with multimorbidity demonstrated varying levels of health literacy and chronic disease self-efficacy. Higher health literacy was independently associated with greater confidence in managing chronic conditions. These findings support the integration of health-literacy-responsive interventions into primary healthcare to strengthen chronic disease self-management and improve person-centred care among older adults with multimorbidity.

## 1. Introduction

For more than a decade, the global population has experienced a rapid increase in the number of older adults living with chronic conditions. These population ageing trends, closely associated with the growing burden of multimorbidity, require multisectoral responses, particularly in health policies, and place healthcare and social care systems under significant pressure, creating numerous challenges [1,2]. In addition, treatment and daily care are essential for older adults with chronic conditions, particularly those living with two or more chronic diseases [3]. Furthermore, effective self-management, which is closely related to health literacy, requires continuous support [4]. Continued care and coordination between primary healthcare and social care services are also essential, particularly in low-resource settings [5].

Studies of patients with multimorbidity in primary healthcare settings have emphasized the importance of patients’ health literacy and knowledge, which influence their confidence and capacity for chronic disease self-management. These factors are particularly important in low-resource settings, where they may inform the development of effective care strategies and interventions for older adults with chronic conditions [4,5].

Increasing longevity has made ageing a significant risk factor for chronic diseases, particularly non-communicable diseases (NCDs) [6]. The most prevalent chronic diseases among older adults include diabetes, cardiovascular diseases, hypertension, respiratory diseases, and cancer, all of which require continuous care and long-term support [7].

A systematic review of the literature found that self-efficacy and greater confidence in chronic disease management among older adults with multimorbidity varies widely and is influenced by both individual skills and social support, particularly in low-resource settings [8]. In addition, patient empowerment plays an important role in chronic disease self-management and is influenced by health literacy, health status, and eHealth literacy [9].

Health literacy refers to individuals’ ability and skills to access, understand, and utilize health information to maintain their health and prevent deterioration of their health status [10]. Health literacy represents an essential component of a broader ecosystem that includes individuals, families, healthcare systems, healthcare providers, social systems, and the environment, representing a key factor in decision-making and collaboration among all actors within the system [11].

It has been found that poor health literacy, particularly among older adults with chronic conditions, is a determinant of informed health decision-making and of patients’ active role in the management of multimorbidity [12]. The factors associated with low health literacy in older adults include socio-demographic factors such as advanced age and educational level; social factors such as family and social support, income, social activities, and socio-economic status; and health-related factors such as chronic conditions, hospitalization, daily functioning (including social and physical activity), frailty, mental health status, and others [13].

Addressing health access equity by improving health literacy, particularly among older adults with chronic conditions in low-resource settings, is essential for building trust in patient–healthcare provider communication, reducing healthcare costs, and improving health outcomes [14]. In addition, evidence suggests that both personal and organizational health literacy empower patients to better understand and use health information, and are fundamental to reducing health inequalities, particularly among older adults with chronic conditions [15].

In low-resource settings, multimorbidity among older adults is associated with poor quality of life, suboptimal disease management, and limited access to integrated multidisciplinary care [16]. In addition, a review study found that shared decision-making, healthcare utilization, and the co-design of care plans with older adults are closely associated with positive self-efficacy and improved care outcomes [17]. Furthermore, a multicentre longitudinal study conducted among older adults with multiple chronic conditions in a middle-income country reported that self-care and self-efficacy behaviours were closely associated with socioeconomic status, educational attainment, and collaboration between patients and caregivers [18]. Although recent studies in low- and middle-income countries have investigated multimorbidity among older adults, they have primarily focused on medication adherence, quality of life, disease management, and self-efficacy. Consequently, evidence regarding the relationship between health literacy and chronic disease self-efficacy remains limited, particularly in primary healthcare settings and low-resource countries [19,20,21,22,23]. Therefore, the present study aimed to examine the association between health literacy and chronic disease self-efficacy among older adults with multimorbidity receiving primary healthcare services in Albania.

## 2. Materials and Methods

### 2.1. Study Setting

The study was conducted in Vlora Municipality, Albania, using randomly selected primary healthcare (PHC) centres. Participants were recruited from PHC centres located in both urban and rural administrative units of the municipality. Place of residence was classified according to the administrative divisions of the 2023 Albanian Population and Housing Census. Urban residence was defined as living in the administrative unit of Vlora (city), whereas rural residence included the administrative units of Novoselë, Orikum, Qendër (Vlora), and Shushicë [24]. According to the 2023 Albanian Population and Housing Census, Vlora Municipality has a population of 83,683 residents, of whom 79.2% reside in urban areas and 20.8% reside in rural areas [24]. To ensure that the study sample reflected the population distribution of the municipality, stratified random sampling was applied, resulting in an estimated sample of 368 urban and 96 rural participants from the total study sample of 464 older adults.

### 2.2. Study Design

A cross-sectional analytical study was conducted among community-dwelling adults aged 65 years or older with at least two diagnosed chronic conditions who were receiving care at primary healthcare (PHC) centres in Vlora Municipality, Albania. Eligible participants were identified from family physician registers and recruited during the study period. Written informed consent was obtained from all participants before data collection. Individuals with diagnosed cognitive impairment (e.g., dementia) or those who declined to participate were excluded from the study.

### 2.3. Data Collection Procedures

Primary healthcare centres were selected using a random sampling procedure. Eligible participants were identified from family physician medical registers at each participating PHC centre. A stratified sampling approach was applied based on participants’ place of residence to ensure that the study sample reflected the urban–rural population distribution within Vlora Municipality. The sampling strata were defined as follows:

- Urban stratum (approximately *n* = 368): Participants residing in the Administrative Unit of Vlora (city).
- Rural stratum (approximately *n* = 96): Participants residing in the administrative units of Novoselë, Orikum, Qendër (Vlora), and Shushicë, with the sample allocated using probability proportional to size (PPS).

Within each stratum and participating PHC centre, eligible participants were selected using simple random sampling from the family physician medical registers. A random selection procedure was used to generate a list of eligible individuals. Participants were then contacted sequentially according to the randomized list until the required sample size for each stratum and PHC centre was achieved. If a selected participant was ineligible or declined to participate, the next eligible individual on the randomized list was invited. This process continued until the target sample size of 464 participants was reached.

### 2.4. Sample Size

The required sample size was calculated using the formula

$$n=z^{2}\frac{p(1-p)}{d^{2}}$$

where

*z* = 1.96 for 95% confidence;

*p* = estimated (0.50);

*d* = margin of error (0.05).

This yielded a minimum required sample size of 384 participants. The final target sample size was increased by approximately 20%, resulting in a total of 464 participants. This adjustment was made to cover for anticipated non-response and ineligibility.

### 2.5. Study Instruments

General Information Questionnaire. A structured questionnaire was developed based on the study objectives and relevant literature to collect participants’ sociodemographic and clinical characteristics. Sociodemographic variables included age, sex, educational level, marital status, employment status, place of residence, and living arrangement (living alone or with family). Clinical information included the primary healthcare (PHC) centre attended, the number and type of chronic diseases, the duration of each chronic condition, self-rated health, and body mass index (BMI).

Health Literacy Questionnaire. Health literacy was assessed using the 16-item European Health Literacy Questionnaire (HLS-EU-Q16) [25]. The HLS-EU-Q16 is a validated instrument that measures individuals’ perceived ability to access, understand, appraise, and apply health-related information across the domains of healthcare, disease prevention, and health promotion. Responses were scored according to the standard HLS-EU-Q16 scoring procedure, with higher scores indicating better health literacy [26].

Chronic Disease Self-Efficacy Questionnaire. Chronic disease self-efficacy was assessed using the Stanford Chronic Disease Self-Efficacy Scale (CDSES-33) [27]. This validated instrument comprises 33 items assessing participants’ confidence in managing different aspects of chronic disease, including exercise, obtaining health information, seeking social support, communicating with healthcare professionals, disease management, household activities, social activities, symptom management, and emotional management. Each item is rated on a 10-point scale ranging from 1 (not at all confident) to 10 (totally confident), with higher scores indicating greater self-efficacy for chronic disease management.

### 2.6. Ethical Considerations

This study was conducted in accordance with the ethical principles of the Declaration of Helsinki and relevant European guidelines for research involving human participants. The study protocol respected participants’ dignity, autonomy, privacy, and confidentiality. Participation was voluntary, and written informed consent was obtained from all participants before data collection commenced. Participants were fully informed about the study objectives, procedures, the type of data to be collected, how the data would be used, measures to ensure data confidentiality, and their right to withdraw from the study at any time without penalty. Confidentiality and the protection of personal data were maintained in accordance with Albanian Law No. 9887, dated 10 March 2008, as amended by Law No. 48/2012 on the Protection of Personal Data. The study protocol was reviewed and approved by the Operator of Health Care Services, the Regional Directorate of Vlora, and the Directorate of Public Health and Health Education. Ethical approval was granted on 3 June 2024 (Approval No. 1084/1). All study procedures were conducted in accordance with the approved protocol.

### 2.7. Statistical Analysis

Statistical analyses were performed using Stata Statistical Software, Version 18 (StataCorp LLC, College Station, TX, USA). Before conducting the main analyses, the dataset was screened for missing data, duplicate observations, implausible values, and compliance with the assumptions underlying the planned statistical procedures. Continuous variables were assessed for normality using the Shapiro–Wilk test, histograms, and normal probability (Q–Q) plots. Given the relatively large sample size (*n* = 464), parametric statistical methods were considered appropriate despite minor deviations from normality because of their robustness in large samples.

Continuous variables are presented as means and standard deviations (SDs), whereas categorical variables are summarized using frequencies and percentages. Health literacy was assessed using the 16-item European Health Literacy Questionnaire (HLS-EU-Q16). Responses were scored according to the standard HLS-EU scoring protocol, whereby responses of “very easy” and “fairly easy” were coded as 1, and “fairly difficult” and “very difficult” were coded as 0. The item scores were summed to produce a total score ranging from 0 to 16, with higher scores indicating better health literacy [25]. For descriptive analyses, health literacy was also categorized into inadequate (0–8), problematic (9–12), and adequate (13–16), in accordance with the HLS-EU-Q16 scoring guidelines [25].

Chronic disease self-efficacy was assessed using the Stanford Chronic Disease Self-Efficacy Scale (CDSES-33) [27]. Participants rated their confidence in managing different aspects of chronic disease on a 10-point Likert scale, with higher scores indicating greater self-efficacy. The internal consistency of the HLS-EU-Q16 and the CDSES-33 was assessed using Cronbach’s alpha coefficient, with values of ≥0.70 considered indicative of acceptable reliability.

Descriptive statistics were used to summarize participants’ sociodemographic and clinical characteristics, health literacy, and chronic disease self-efficacy. Independent-samples *t*-tests and one-way analysis of variance (ANOVA) were performed to compare mean chronic disease self-efficacy scores across participant characteristics, as appropriate. Pearson’s correlation coefficients were calculated to examine the strength and direction of associations between health literacy, chronic disease self-efficacy, and selected continuous variables.

Hierarchical multiple linear regression analysis was performed to examine the independent association between health literacy and chronic disease self-efficacy after adjustment for demographic and clinical characteristics. Three hierarchical models were constructed based on theoretical and clinical relevance. Model 1 included demographic variables (age, sex, and educational level), Model 2 additionally included clinical variables (body mass index, self-rated health, and number of chronic diseases), and Model 3 further included health literacy. Regression assumptions, including linearity, normality of residuals, homoscedasticity, multicollinearity, and influential observations, were assessed using standard diagnostic procedures before interpretation of the final model. Results are presented as unstandardized regression coefficients (B), standardized regression coefficients (β), 95% confidence intervals (CIs), and corresponding *p*-values. All statistical tests were two-sided, and a two-sided *p*-value < 0.05 was considered statistically significant.

## 3. Results

### 3.1. Participant Characteristics

A total of 464 community-dwelling older adults with multimorbidity participated in the study. The mean age was 69.93 ± 4.72 years (range: 65–84 years), and the mean body mass index (BMI) was 26.48 ± 4.58 kg/m^2^ (range: 17.58–57.21 kg/m^2^). Women comprised 54.3% (*n* = 252) of the study population, while 45.7% (*n* = 212) were men. More than half of the participants (52.4%) had completed primary education, 36.4% had secondary education, and 11.2% had tertiary education. Most participants were married or living with a partner (83.6%), whereas 10.6% were widowed and 5.8% were single, divorced, or separated. Regarding employment status, 41.2% were retired, 33.8% remained employed, and 25.0% were unemployed or economically inactive.

Participants were distributed across urban (44.8%), peri-urban (10.8%), and rural (44.4%) areas. The largest religious group was Muslim (58.4%), followed by Orthodox Christian (32.1%). Self-rated health was reported as good by 69.0% of participants, very good by 15.1%, fair by 12.9%, excellent by 2.8%, and poor by 0.2%. In accordance with the study inclusion criteria, 89.7% of participants reported living with two chronic diseases, whereas 10.3% reported three chronic diseases. According to the World Health Organization BMI classification, 42.0% had normal body weight, 38.8% were overweight, 18.3% were obese, and 0.9% were underweight. Based on the HLS-EU-Q16 classification, 42.9% of participants had adequate health literacy, 25.4% had problematic health literacy, and 31.7% had inadequate health literacy (Table 1).

### 3.2. Chronic Disease Profile

The prevalence of chronic conditions reported by the participants is presented in Table 2. Hypertension was the most frequently reported chronic condition (72.6%), followed by diabetes mellitus (70.3%). Chronic heart failure (11.9%), rheumatic diseases (11.4%), chronic obstructive pulmonary disease (8.8%), and chronic kidney disease (6.2%) were also commonly reported. Anemia affected 4.3% of participants, whereas neurological, gastrointestinal, hepatic, mental health, and oncological conditions were reported less frequently, each affecting fewer than 3% of the study population (Table 2).

### 3.3. Descriptive Statistics of Health Literacy and Chronic Disease Self-Efficacy

Descriptive statistics for the principal study variables are presented in Table 3. The mean health literacy score measured using the HLS-EU-Q16 was 10.69 ± 4.79, with scores ranging from 0 to 16. The median score was 12. The mean overall chronic disease self-efficacy score was 5.56 ± 1.78, with observed values ranging from 1.00 to 10.00. Across the individual self-efficacy domains, the highest mean scores were observed for physician communication (6.38 ± 2.18) and health information seeking (6.19 ± 2.52), whereas the lowest mean scores were observed for exercise (4.59 ± 2.21) and symptom management (5.00 ± 2.05) (Table 3).

### 3.4. Reliability of the Study Instruments

The internal consistency of the study instruments was evaluated using Cronbach’s alpha coefficient. The HLS-EU-Q16 demonstrated excellent internal consistency (Cronbach’s α = 0.924). Similarly, the overall Stanford Chronic Disease Self-Efficacy Scale (CDSES-33) demonstrated excellent reliability (Cronbach’s α = 0.974). Reliability coefficients for the individual CDSES domains ranged from 0.840 to 0.958, indicating good to excellent internal consistency across all multi-item domains (Table 4).

### 3.5. Bivariate Analysis

Bivariate analyses were conducted to examine differences in chronic disease self-efficacy according to participants’ demographic and clinical characteristics. No statistically significant difference in mean self-efficacy was observed between men and women (*p* = 0.280). In contrast, educational attainment was significantly associated with chronic disease self-efficacy (*p* = 0.013), with participants who had completed tertiary education reporting the highest mean scores.

Self-rated health was also significantly associated with chronic disease self-efficacy (*p* < 0.001). Participants reporting excellent or very good health had higher mean self-efficacy scores than those reporting fair or poor health. Participants with two chronic diseases reported significantly higher self-efficacy than those with three chronic diseases (*p* < 0.001). Significant differences were also observed across health literacy categories (*p* < 0.001), with mean self-efficacy scores increasing from inadequate to problematic and adequate health literacy (Table 5).

### 3.6. Correlation Analysis

Pearson’s correlation analysis demonstrated a moderate positive correlation between health literacy and overall chronic disease self-efficacy (*r* = 0.407, *p* < 0.001). Positive correlations were observed between health literacy and all domains of chronic disease self-efficacy, with correlation coefficients ranging from 0.196 to 0.437.

Age showed weak positive correlations with both health literacy and chronic disease self-efficacy, whereas the number of chronic diseases was negatively correlated with both variables. Body mass index was not significantly correlated with either health literacy or chronic disease self-efficacy (Table 6).

### 3.7. Hierarchical Multiple Linear Regression Analysis

Hierarchical multiple linear regression was performed to determine whether health literacy independently predicted chronic disease self-efficacy after adjustment for demographic and clinical characteristics. The inclusion of health literacy in the final model increased the proportion of explained variance from 15.1% to 24.3%.

In the fully adjusted model, health literacy was independently associated with chronic disease self-efficacy (B = 0.123, 95% CI: 0.090–0.155; β = 0.331; *p* < 0.001). Age and tertiary education remained significant predictors, whereas poorer self-rated health was associated with lower self-efficacy. After adjustment for health literacy, the number of chronic diseases was no longer statistically significant (Table 7).

### 3.8. Regression Diagnostics

Diagnostic analyses indicated that the assumptions of the final regression model were adequately satisfied. No evidence of problematic multicollinearity was observed (mean variance inflation factor = 2.54). The Breusch–Pagan test indicated no evidence of heteroscedasticity (*p* = 0.356), while inspection of residual plots supported the assumptions of linearity and normality. Cook’s distance values indicated that no individual observation exerted undue influence on the regression model (Table 8).

### 3.9. Sensitivity Analysis

A sensitivity analysis was conducted by replacing the continuous health literacy score with categorical health literacy levels in the regression model. The findings were consistent with those of the primary analysis. Compared with participants with inadequate health literacy, those with problematic and adequate health literacy demonstrated significantly higher chronic disease self-efficacy. The explanatory power of the sensitivity model (R^2^ = 0.240) was comparable to that of the primary regression model (R^2^ = 0.243), supporting the robustness of the findings (Table 9).

## 4. Discussion

The present study examined the association between health literacy and chronic disease self-efficacy among community-dwelling older adults with multimorbidity receiving primary healthcare services in Vlora Municipality, Albania. The findings demonstrated that higher health literacy was independently associated with greater chronic disease self-efficacy after adjustment for demographic and clinical characteristics. Educational attainment and self-rated health were also significant predictors of self-efficacy, whereas age showed a modest positive association. These findings contribute to the growing evidence that health literacy is an important determinant of chronic disease self-management among older adults with multimorbidity and provide new evidence from a primary healthcare setting in Albania, where research in this area remains limited.

Women represented a slightly higher proportion of the study sample (54.3%) than men (45.7%) while the study population was predominantly composed of older adults living with hypertension and diabetes mellitus. This distribution is consistent with previous studies reporting a higher prevalence of multimorbidity among older women than men [28,29]. Meanwhile the epidemiological profile of multimorbidity found has similar patterns reported in low- and middle-income countries [30]. Furthermore, these findings are consistent with previous studies showing that cardiovascular and metabolic diseases are the most frequent chronic conditions among older adults and often coexist because they share common behavioural, metabolic, and age-related risk factors [31,32,33]. The high prevalence of these conditions highlights the growing need for integrated, person-centred chronic disease management strategies within primary healthcare [34,35,36].

A major finding of the present study was the high prevalence of limited health literacy among older adults with multimorbidity. Although a substantial proportion of participants demonstrated adequate health literacy, many were classified as having inadequate or problematic health literacy. These findings are consistent with previous European studies reporting that older adults experience greater difficulty accessing, understanding, appraising, and applying health information because of age-related cognitive changes, multiple chronic conditions, complex treatment regimens, and lower educational attainment [37,38,39].

Limited health literacy has consistently been associated with poorer medication adherence, reduced use of preventive services, increased healthcare utilization, and poorer clinical outcomes among individuals with multimorbidity [40,41].

The principal finding of this study was that health literacy remained an independent predictor of chronic disease self-efficacy after adjustment for demographic and clinical characteristics. Older adults with higher health literacy reported greater confidence in managing their chronic conditions, supporting previous evidence that health literacy is a fundamental component of effective self-management. Individuals who are able to obtain, understand, evaluate, and apply health information are more likely to adhere to treatment recommendations, communicate effectively with healthcare professionals, and make informed decisions regarding disease management [42,43,44]. These findings are consistent with previous studies demonstrating that health literacy contributes to improved self-efficacy and enhances patients’ ability to manage chronic conditions successfully [45,46,47,48,49]. Meanwhile, recent multicentre evidence has further suggested that health literacy should be considered within the broader context of patient and care complexity among older adults with multimorbidity. In particular, inadequate health literacy and higher care complexity may interact synergistically, increasing vulnerability to adverse health outcomes and creating additional challenges for chronic disease management. Although care complexity was not assessed in the present study, our findings are consistent with the view that health literacy represents an important component influencing patients’ capacity to manage chronic conditions [50].

Educational attainment remained significantly associated with chronic disease self-efficacy in the adjusted analyses. Participants with higher educational attainment demonstrated greater confidence in managing their chronic conditions, a finding that is consistent with previous research linking education with improved health literacy, greater access to health information, and more effective self-management behaviours [48,49]. Likewise, participants reporting poorer self-rated health had significantly lower self-efficacy scores, suggesting that perceived health status influences individuals’ confidence in managing chronic illness. Similar associations have been reported among older adults with multimorbidity in previous studies [51,52].

The findings have important implications for primary healthcare practice. Given the high prevalence of limited health literacy observed in this study, healthcare professionals should routinely assess health literacy when caring for older adults with multimorbidity. Interventions such as plain-language communication, the teach-back method, individualized education, and structured self-management programmes may improve patients’ understanding of treatment recommendations and strengthen their confidence in managing chronic conditions [53,54,55,56]. Nurses, family physicians, and other primary healthcare professionals play a central role in delivering person-centred care and supporting self-management among older adults with multimorbidity [57,58].

This study has several strengths. It is among the first studies conducted in Albania to examine the association between health literacy and chronic disease self-efficacy among older adults with multimorbidity using validated instruments. The use of stratified random sampling, validated questionnaires, and multivariable regression analysis strengthens the reliability and validity of the findings.

Several limitations should be considered when interpreting the findings. First, the cross-sectional design precludes conclusions regarding causal relationships between health literacy and chronic disease self-efficacy. Second, data were collected using self-reported questionnaires, which may be subject to recall and social desirability bias. Third, participants were recruited from a single municipality in Albania, which may limit the generalizability of the findings to other settings. Finally, although validated instruments were used to assess health literacy and chronic disease self-efficacy, future longitudinal and multicentre studies are needed to confirm these findings and examine changes over time. Future research should also incorporate measures of patient and care complexity to determine whether these factors modify or mediate the relationship between health literacy and chronic disease self-efficacy and to further inform the development of person-centred chronic disease management strategies.

## 5. Conclusions

This study provides evidence that health literacy is an important determinant of chronic disease self-efficacy among older adults with multimorbidity receiving primary healthcare services. Higher health literacy was independently associated with greater confidence in managing chronic conditions, even after adjustment for demographic and clinical factors. These findings highlight the importance of incorporating health literacy assessment and tailored communication strategies into person-centred primary healthcare approaches for older adults with multimorbidity. Beyond its role as an individual capacity, health literacy may represent an important component of the broader vulnerability profile of older adults with complex care needs. Future longitudinal and multicentre studies should investigate how health literacy interacts with other dimensions of patient and care complexity, including clinical burden, functional limitations, and healthcare demands, to better identify individuals at increased risk of poor self-management and adverse outcomes. Such evidence may support the development of more targeted, integrated, and patient-centred interventions aimed at strengthening self-efficacy and improving chronic disease management among older adults with multimorbidity.

## Figures and Tables

**Table 1:** Sociodemographic and clinical characteristics (*n* = 464).

Characteristic	Category	*n*	%
Gender	Male	212	45.7
	Female	252	54.3
Education	Primary	243	52.4
	Secondary	169	36.4
	Tertiary	52	11.2
Marital status	Married/Living with partner	388	83.6
	Single/Divorced/Separated	27	5.8
	Widowed	49	10.6
Employment status	Employed	157	33.8
	Unemployed/Economically inactive	116	25
	Retired	191	41.2
Residence	Urban	208	44.8
	Peri-urban	50	10.8
	Rural	206	44.4
Religion	Orthodox Christian	149	32.1
	Muslim	271	58.4
	Catholic	30	6.5
	Other	14	3
Self-rated health	Excellent	13	2.8
	Very good	70	15.1
	Good	320	69
	Fair	60	12.9
	Poor	1	0.2
Number of chronic diseases	Two	416	89.7
	Three	48	10.3
BMI category	Underweight	4	0.9
	Normal weight	195	42
	Overweight	180	38.8
	Obese	85	18.3
Health literacy category	Inadequate	147	31.7
	Problematic	118	25.4
	Adequate	199	42.9
**Continuous variables**	**Mean ± SD**	**Range**	
Age (years)	69.93 ± 4.72	65–84	
Body Mass Index (kg/m^2^)	26.48 ± 4.58	17.58–57.21	

**Table 2:** Prevalence of chronic conditions reported by the study participants (*n* = 464).

Condition	*n*	%
Cardiovascular		
Hypertension	337	72.6
Chronic heart failure	55	11.9
Hypercholesterolemia	8	1.7
Atherosclerosis	7	1.5
Endocrine/Metabolic		
Diabetes mellitus	326	70.3
Hyperthyroidism	5	1.1
Obesity	2	0.4
Respiratory		
COPD (chronic obstructive pulmonary disease)	41	8.8
Rheumatologic/Musculoskeletal		
Rheumatic diseases	53	11.4
Systemic lupus erythematosus	1	0.2
Gout	1	0.2
Lumbar disc herniation	1	0.2
Renal/Urologic		
Chronic kidney disease	29	6.2
Benign prostatic hyperplasia	4	0.9
Chronic cholecystitis	1	0.2
Hematologic		
Anemia	20	4.3
Thalassemia	5	1.1
Gastrointestinal/Hepatic		
Liver cirrhosis	12	2.6
Gastrointestinal diseases (other)	7	1.5
Hepatitis	6	1.3
Chronic pancreatitis	2	0.4
Neurological		
Parkinson’s disease	8	1.7
Epilepsy	7	1.5
Migraine	7	1.5
Multiple sclerosis	5	1.1
Mental health/Cognitive		
Mental health disorders (other)	11	2.4
Alzheimer’s disease	6	1.3
Schizophrenia	2	0.4
Oncological		
Melanoma	1	0.2

**Table 3:** Descriptive statistics of continuous study variables (*n* = 464).

Variable	*n*	Mean	SD	Median	Minimum	Maximum
Age (years)	464	69.93	4.72	68	65	84
Body Mass Index (kg/m^2^)	464	26.48	4.58	25.82	17.58	57.21
Health literacy score (HLS-EU-Q16)	464	10.69	4.79	12	0	16
CDSE Total Score	464	5.56	1.78	5.52	1	10
CDSE Exercise	464	4.59	2.21	4.33	1	10
CDSE Health Information	463	6.19	2.52	6	1	10
CDSE Social Support	464	5.9	2.25	6	1	10
CDSE Physician Communication	464	6.38	2.18	6.67	1	10
CDSE Disease Management	464	5.54	1.94	5.6	1	10
CDSE Household Activities	464	5.77	2.39	5.67	1	10
CDSE Social Activities	464	5.32	2.2	5	1	10
CDSE Symptom Management	464	5	2.05	5	1	10
CDSE Emotional Management	464	5.33	2.07	5.33	1	10

**Table 4:** Internal consistency reliability of the study instruments.

Scale/Domain	Number of Items	Cronbach’s α	Interpretation
HLS-EU-Q16 Total Scale	16	0.924	Excellent
CDSES-33 Total Scale	33	0.974	Excellent
Exercise	3	0.925	Excellent
Health Information	1	—	Single-item measure
Social Support	4	0.84	Good
Physician Communication	3	0.875	Good
Disease Management	5	0.921	Excellent
Household Activities	3	0.945	Excellent
Social Activities	2	0.876	Good
Symptom Management	6	0.955	Excellent
Emotional Management	6	0.958	Excellent

**Table 5:** Bivariate comparisons of chronic disease self-efficacy according to participant characteristics (*n* = 464).

Variable	Category	Mean ± SD	Test Statistic	*p*-Value
Gender	Male	5.46 ± 1.71	t = −1.08	0.28
	Female	5.64 ± 1.84		
Education	Primary	5.35 ± 1.83	F = 4.36	0.013
	Secondary	5.69 ± 1.55		
	Tertiary	6.08 ± 2.06		
Residence	Urban	5.38 ± 1.76	F = 2.95	0.053
	Peri-urban	5.38 ± 1.59		
	Rural	5.78 ± 1.83		
Self-rated health	Excellent	7.02 ± 2.30	F = 8.26	<0.001
	Very good	6.27 ± 1.56		
	Good	5.48 ± 1.69		
	Fair	4.84 ± 1.96		
	Poor	3.86		
Number of chronic diseases	Two	5.68 ± 1.75	F = 18.56	<0.001
	Three	4.53 ± 1.71		
Health literacy category	Inadequate	4.59 ± 1.70	F = 43.61	<0.001
	Problematic	5.61 ± 1.51		
	Adequate	6.25 ± 1.66		

**Table 6:** Pearson correlations between Health Literacy and Chronic Disease Self-Efficacy and selected continuous variables.

Variable	*r*	*p*-Value
CDSE Total	0.407	<0.001
Exercise	0.196	<0.001
Information	0.282	<0.001
Support	0.437	<0.001
Doctor communication	0.368	<0.001
Disease management	0.341	<0.001
Home management	0.329	<0.001
Social activities	0.331	<0.001
Symptom management	0.3	<0.001
Emotional management	0.383	<0.001
Age	0.141	0.002
BMI	0.048	0.298
Number of chronic diseases	−0.270	<0.001

**Table 7:** Hierarchical multiple linear regression predicting Chronic Disease Self-Efficacy (*n* = 464).

Predictor	Model 1 B (95% CI)	Model 2 B (95% CI)	Model 3 B (95% CI)
Age	0.081 ***	0.073 ***	0.058 ***
Female	NS	NS	NS
Secondary education	0.422 *	0.349 *	NS
Tertiary education	0.807 **	0.785 **	0.556 *
BMI	—	NS	NS
Self-rated health (Good)	—	−1.456 **	−1.174 **
Self-rated health (Fair)	—	−1.955 ***	−1.372 **
Self-rated health (Poor)	—	−3.535 *	−3.747 *
Three chronic diseases	—	−0.807 **	NS
Health literacy score	—	—	0.123 *
Model R^2^	0.067	0.151	0.243
Adjusted R^2^	0.059	0.132	0.225
F statistic	8.28 *	8.03 *	13.18 *

Note: NS, not significant; * *p* < 0.05; ** *p* < 0.01; *** *p* < 0.001.

**Table 8:** Regression diagnostic statistics for the final hierarchical regression model.

Diagnostic Assessment	Result	Interpretation
Mean Variance Inflation Factor (VIF)	2.54	No evidence of problematic multicollinearity
Range of VIF values	1.02–8.27	All values below accepted threshold (<10)
Breusch–Pagan test	χ^2^ = 0.85, *p* = 0.356	Homoscedasticity assumption satisfied
Residual histogram	Approximately normal	Acceptable normality
Normal Q–Q plot	Minor tail deviations	Acceptable normality for regression
Residual versus fitted plot	Random scatter	Linearity and constant variance supported
Mean Cook’s distance	0.0033	Very low influence of individual observations
Maximum Cook’s distance	0.544	No influential observations identified

**Table 9:** Sensitivity analysis of the association between Health Literacy categories and Chronic Disease Self-Efficacy (*n* = 464).

Predictor	B	95% CI	*p*-Value
Age	0.059	0.028 to 0.091	<0.001
Female	0.165	−0.128 to 0.458	0.269
Secondary education	0.189	−0.128 to 0.506	0.242
Tertiary education	0.602	0.119 to 1.085	0.015
BMI	0.011	−0.021 to 0.042	0.499
Good self-rated health	−1.19	−2.082 to −0.297	0.009
Fair self-rated health	−1.493	−2.467 to −0.519	0.003
Poor self-rated health	−3.652	−6.872 to −0.431	0.026
Three chronic diseases	−0.364	−0.875 to 0.146	0.162
Problematic health literacy	0.817	0.426 to 1.208	<0.001
Adequate health literacy	1.335	0.973 to 1.696	<0.001

## Data Availability

The data presented in this study are available on request from the corresponding author regarding privacy restrictions.

## Abbreviations

The following abbreviations are used in this manuscript:

HLS-EU-Q16	Health Literacy Survey European Union Questionnaire (short form, 16 items)
QoL	Quality of life
NCDs	Non-communicable diseases
PHC	Primary healthcare
COPD	Chronic obstructive pulmonary disease
BMI	Body mass index
